# Evaluation of Injectable Constructs for Bone Repair with a Subperiosteal Cranial Model in the Rat

**DOI:** 10.1371/journal.pone.0071683

**Published:** 2013-08-13

**Authors:** Marta Kisiel, Agnieszka S. Klar, Mikaël M. Martino, Manuela Ventura, Jöns Hilborn

**Affiliations:** 1 Division of Polymer Chemistry, Department of Chemistry-Ångström, Sciences Life Laboratory, Uppsala University, Uppsala, Sweden; 2 Medical Faculty, University Hospital Akademiska, Uppsala, Sweden; 3 Tissue Biology Research Unit, Department of Surgery, University Children’s Hospital, Zurich, Switzerland; 4 Laboratory of Host Defense, WPI Immunology Frontier Research Center (IFReC), Osaka University, Osaka, Japan; 5 Biomaterials, Radboud University, Nijmegen Medical Centre, Nijmegen, The Netherlands; University of Minho, Portugal

## Abstract

While testing regenerative medicine strategies, the use of animal models that match the research questions and that are related to clinical translation is crucial. During the initial stage of evaluating new strategies for bone repair, the main goal is to state whether the strategies efficiently induce the formation of new bone tissue at an orthotopic site. Here, we present a subperiosteal model in rat calvaria that allow the evaluation of a broad range of approaches including bone augmentation, replacement and regeneration. The model is a fast to perform, minimally invasive, and has clearly defined control groups. The procedure enables to evaluate the outcomes quantitatively using micro-computed tomography and qualitatively by histology and immunohistochemistry. We established this new model, using bone morphogenetic protein-2 as an osteoinductive factor and hyaluronic acid hydrogel as injectable biomaterial. We showed that this subperiosteal cranial model offers a minimally invasive and promising solution for a rapid initial evaluation of injectables for bone repair. We believe that this approach could be a powerful platform for orthopedic research and regenerative medicine.

## Introduction

Non-union fractures and traumatic bone defects represent severe medical and socio-economic problems. Currently, the gold standard for the treatment of such bone defects is the implantation of autologous bone grafts harvested from the patient’s iliac crest [1]. While effective, this method is associated with donor site morbidity and limitations in the amount of bone that can be used [1], [2]. Therefore, an overarching aim in orthopedics is the development of alternative regenerative medicine therapies that lead to bone augmentation.

Standards for the biological evaluation of new treatment modalities, established by the International Organization for Standardization (ISO) 10993, include the evaluation of a number of variables that must be evaluated before a product can be considered safe and efficient for human use. At the moment, the development of regenerative medicine strategies for bone tissue from concept to product takes 4–10 years from concept to product and involves a cost of 5–300 million dollars, depending on the regulatory process required [3]. As part of this development process, an evaluation of the approach’s safety and biocompatibility and the associated immunological response, as well as its ability to induce bone formation, can only be achieved *in vitro* [4] and in animal models [5], [6].

As yet, there is no rapid and simple preclinical model to allow the evaluation of regenerative strategies for bone. The use of large animals such as sheep is usually required before clinical trials, since these animals display characteristics of bone anatomy and biology that are close to those of human [7], [8]. However, the cost and time course of using large animals are not suitable for the screening and the rapid testing of new strategies. On the other hand, small animals, and in particularly rats, are easier to handle and more suitable for initial testing [9]. With rats, outcomes can be determined after a short period of time, and the variation between individuals of the same strain, age, gender, and weight is very small [10]. The handling of the animals is relatively easy and low-cost, since they are tolerant of surgery [11]. Finally, it is possible to use advanced functional imaging techniques for rats that are not available for large animals [4]. For these and other reasons, rats are currently the most commonly animal used for musculoskeletal research (45% of animal research) [11].

In our laboratory, we have gained experience with various animal models, including the ectopic bone formation model [12], [13] and various defect models [14]. As a standard and a first approach, the ectopic model is often widely used by researchers in order to evaluate the osteoinductive capacity of cells, morphogens, and biomaterials [15]. However, while simple and fast, this model has a drawback in that the formation of bone occurs in an extra-osseous site. Therefore, the ectopic bone model is not representative of the clinical needs. The defect models are much more clinically relevant, although the size of the defect matters, since defects that are not large enough heal spontaneously [16]. For example, critical-size defect models at orthotopic sites such as the tibia are very relevant, but they are complicated and can be associated with a high failure rate. Moreover, additional stabilization by heavy fixation devices is often required, and this may damage surrounding soft tissues and change biomechanical conditions are often required [3]. A simpler and more relevant model to illustrate the potential of clinical translation potential is that of calvarial defects in the rat [3], [17]. However, the surgery is still relatively long and invasive, and it can yield to non-negligible variability as the result of bleeding. In addition, as in all defect models, postoperative treatment with painkillers is required for several days. Thus, when the defect models are used, the long surgical intervention and the large injury expose animals to an extremely stressful situation and to postoperative pain that should be treated with painkillers. However, it is known that both stress and the use of painkillers can modify the immune response and strongly influence bone healing [18]–[20]. In addition, clinical applications for constructs inducing bone formation are not limited to repair defects. For instance, one of the main clinical applications is spinal fusion [15], in which no defects need to be repaired.

Recently, there has been an interest in using the periosteal space of the bone as the site for biomaterial testing, since this cellular membrane covering the bone is rich in progenitor cells and various cytokines [21], [22]. For instance, Eryilmaz *et* al. obtained bone formation while implanting demineralized bone matrix (DBM) [23]. However, this method required an elevation of the periosteal flaps and their suturing. In contrast, Fujimoto *et* al. induced bone in the subperiosteal space by less invasive injections of transforming growth factor-β (TGF-β) [24]. Nevertheless, in this study multiple injections were required because TGF-β was applied without a scaffold. In contrast, Stevens *et* al. used an injectable biomaterial that was placed under the periosteal of the tibia in rabbits and triggered bone formation [25]. Another interesting study engineered cartilage in the same way [26].

Recently, we have presented a model in which a bone-inducing construct, a hyaluronic acid (HA) hydrogel loaded with bone morphogenetic protein-2 (BMP-2), was tested in the periosteal space of the mandible [6]. The benefit of using HA hydrogel as a scaffold for BMP-2 is that it becomes a solid mass after mixing. Also, implantation by injection makes the intervention shorter and minimally invasive, and therefore it is easier and less stressful for the animal. However, we found a periosteal mandibular model to be specifically relevant for alveolar bone augmentation. Therefore, we now present a new subperiosteal model in the cranial bone of the rat. The cranial bone is more accessible than mandible, and the intervention can be controlled visually. This model allowed us to rapidly evaluate the osteogenic potential of injectable constructs.

## Results

### 1. Surgical Intervention

**Figure 1:**
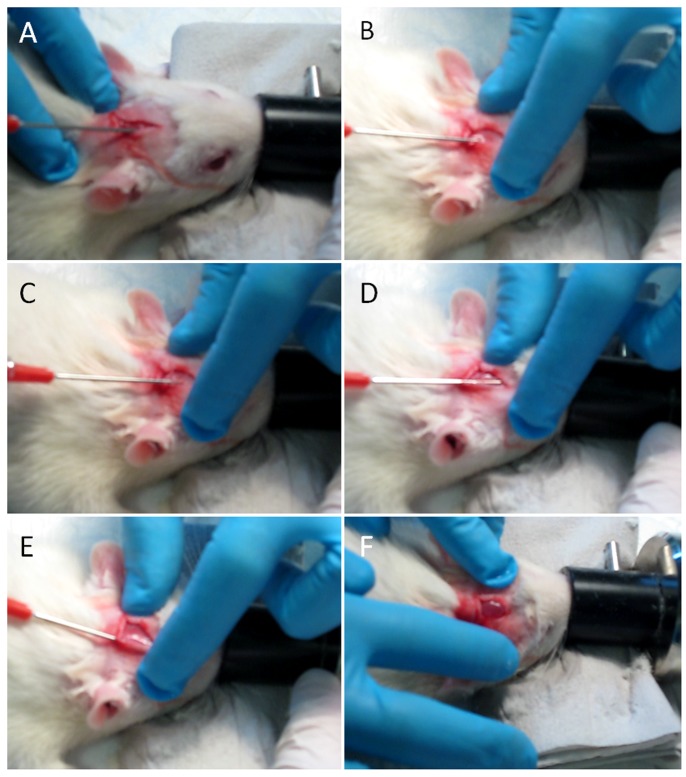
Key aspects of the surgical procedure. (A, B) The top of a blunt needle is inserted at an angle of 45° into the periosteal space of the cranial bone and (C) is pushed forward with an angle of 15–20° onto the bone surface towards the face plane. The length of the needle insertion into the subperiosteal space is visually controlled. After it reaches 1 cm, the tip is moved (D) to the left and (E) to the right, in order to make space for an injectable construct. (F) The construct is injected in a volume of 200 µL, forming a small bump.

HA hydrogels without BMP-2 or containing a high (30 µg) or low (1 µg) dose of BMP-2 were prepared. The constructs or saline (sham group) were then injected into the periosteal spaces of rat cranial bones (Fig. 1 A–F). From anesthesia induction to the skin closure, the surgical intervention lasted approximately 15 min. The procedure was easy to perform by one surgeon, and all constructs were injected at a desired site (Video S1). The periosteum was only punctured once, and no sutures were required to close it. No rupture of the periosteum or other perioperative complications were observed. Postoperative recovery was uneventful, and the animals resumed normal ambulation without any sign of pain or distress during the study period. The wounds healed normally and were covered with hairs. No abnormal behaviour was noted during the 6-week duration of the study.

### 2. Efficacy of New Bone Formation

**Figure 2:**
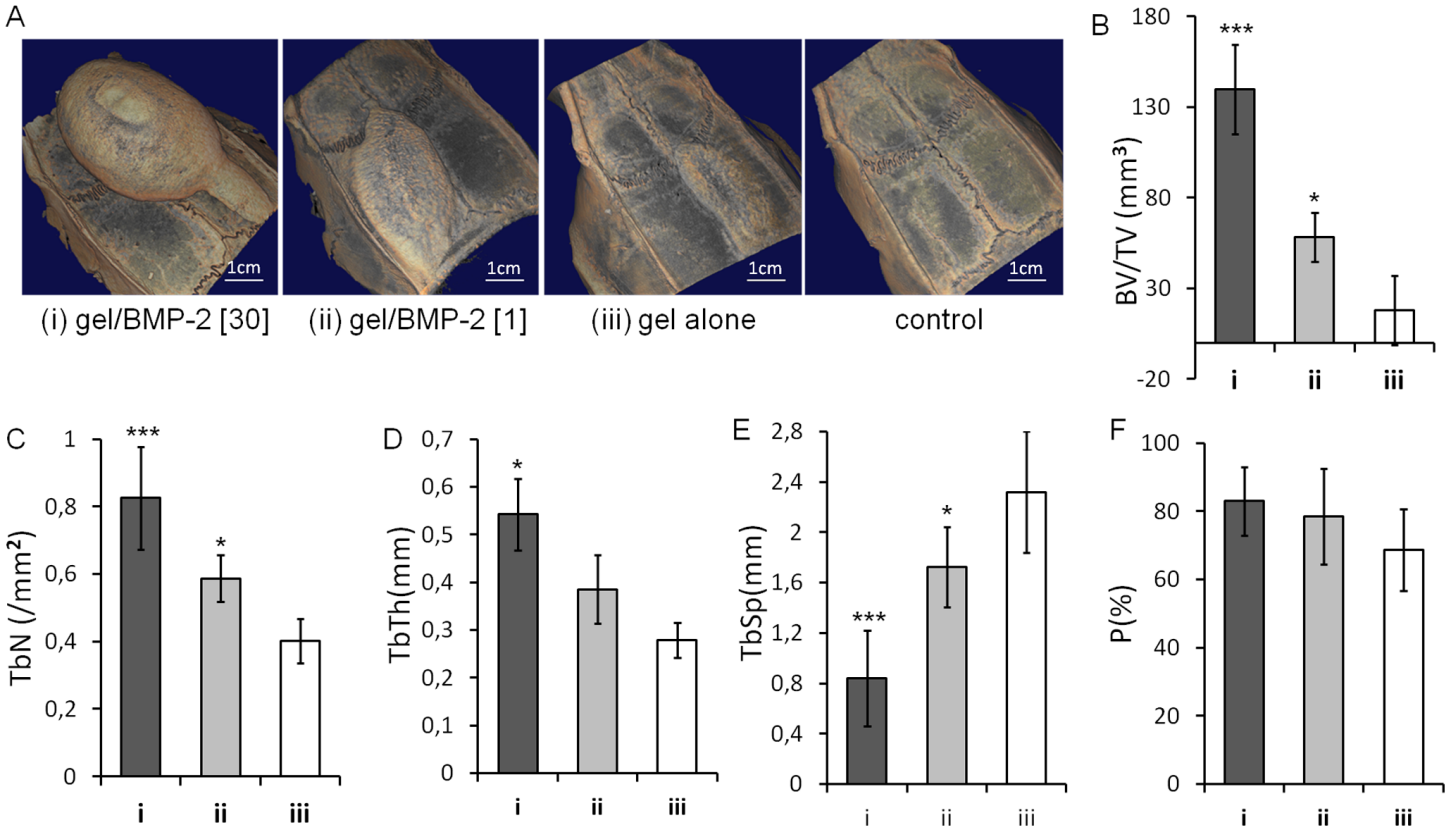
New bone formation. (A) Representative 3D micro-CT reconstruction of cranial bone treated with (i) gel containing 30 µg of BMP-2, (ii) gel containing 1 µg of BMP-2, (iii) gel without BMP-2, or saline (control) at 6 weeks post-injection. Scale bar is 1 cm. MicroCT analysis of (B) the trabecular bone volume (BV/TV [mm^3^]), (C) trabecular number (TbN [/mm^2^]), (D) trabecular thickness (TbTh [mm]), (E) trabecular separation (TbSp [mm]), and (F) trabecular porosity (P [%]). Shown are means ± SD, n = 3. Comparisons between gel groups and the control group were made by two tailed Student’s *t*-test for paired samples (*p<0.05; ***p<0.001).

After 6 weeks, the rats were sacrificed, and the cranial bones were harvested for further evaluation. The volume of new bone formed was determined by micro-computed tomography (micro-CT) (Fig 2 A). Quantitative analysis was performed by comparing the bone volume of specimens from the rats treated with constructs to those from animals treated with saline. HA hydrogels that did not contain BMP-2 (gel alone) showed only a slight bone ingrowth (18 mm^3^) (Fig. 2 A and B). In contrast, HA hydrogels with either the high or low dose of BMP-2 were able to cause significant bone ingrowth (*p*<0.05): 139 mm^3^ and 57 mm^3^, respectively. No statistically significant difference was found between the gel alone and the gel with the low dose of BMP-2. In addition, in order to determine the degree of correlation between the volume of the new bone formed and the concentration of BMP-2, we determined the non-parametric Spearman’s correlation coefficient, and found a significant correlation between both parameters (p<0.001). Furthermore, the trabecular number (Fig. 2 C), thickness (Fig. 2 D) and separation (Fig. 2 E) of bones formed by the gels with BMP-2 were significantly higher than the bone induced by gel alone (Fig. 2). There was only slight difference in the bone porosity between three groups (Fig. 2 F).

### 3. New Bone Morphology

**Figure 3:**
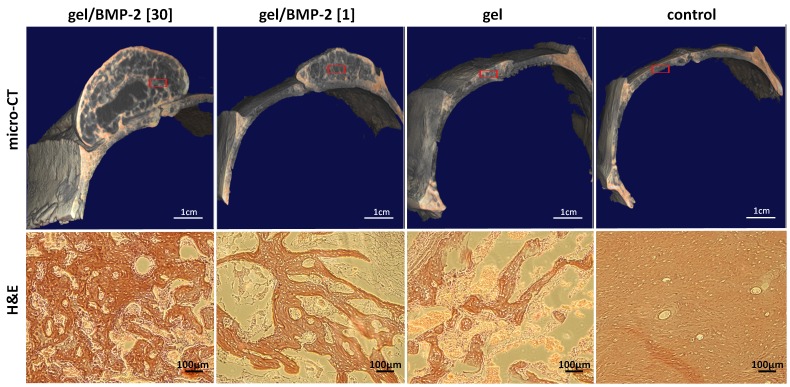
Morphology of the new bone formed. Representative transversal micro-CT reconstructions and cross-sections stained with H&E of cranial bone treated with gel containing 30 µg of BMP-2, gel containing 1 µg of BMP-2, gel without BMP-2, or no gel (saline control) at 6 weeks post-injection. The red square on the micro-CT reconstructions represents the area stained with H&E.

Histological assessment of calvarial bone composition at 6 weeks post-injection was performed on H&E-stained sections to provide information about the quality and morphologic characteristics of the newly formed bone tissue. Representative histological images from each group are shown in Fig. 3. Calvarias injected with HA hydrogel with a high dose of BMP-2 showed the largest amount of bone ingrowth (Fig. 3). Within the new bone formed, we observed structures resembling trabecular bone with bone marrow spaces. Woven bone with a weak trabecular structure and large bone marrow spaces was formed in the groups receiving HA hydrogel containing the lower dose of BMP-2. In this group, more ossification appeared in the periphery than in the center of the new bone. As expected, the group without BMP-2 had a minimal amount of bone formation. In all groups, there was no histological sign of the presence of inflammatory cells or fibrous tissue. Moreover, we did not observe cartilage or residual HA hydrogel at the injection site (data not shown).

**Figure 4:**
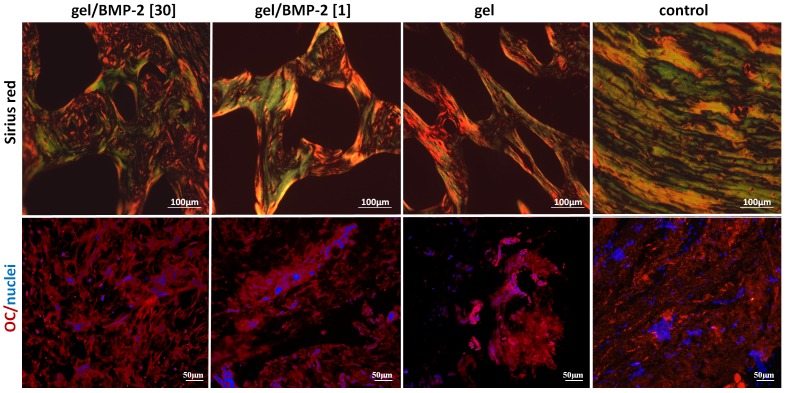
Quality of the new bone formed. Representative cross sections of cranial bone treated with gel containing 30 µg of BMP-2, gel containing 1 µg of BMP-2, gel without BMP-2, or no gel (saline control) at 6 weeks post-injection. The specimens were stained with Sirius red to show collagen fiber orientation and were also stained for OC (in red). Nuclei are in blue. The green-yellowish color represents organized collagen fibers.

As an indicator of the quality of the new bone formed, we examined the assembly of collagen fibers using Sirius red staining and detected the presence of osteoclacin (OC), by immunostaining (Fig. 4). We found collagen fibers of the highest degree organization, in the group containing the higher concentration of BMP-2, when compared to those receiving less (or no) BMP-2. Furthermore, in all experimental groups, positive signals for OC were detected, at the sites at which tissue ingrowth was identified. The deposition of bone matrix in the bone tissues formed by the gels containing 30 µg of BMP-2 was higher than that in the bone tissue derived from the gels containing 1 µg or no BMP-2 (Fig. 4). As a control, we used areas of native calvarial bone which were positive for OC. The negative control (treated only with secondary antibody) showed no positive staining (data not shown).

### 4. Angiogenesis within the New Bone Formed

**Figure 5:**
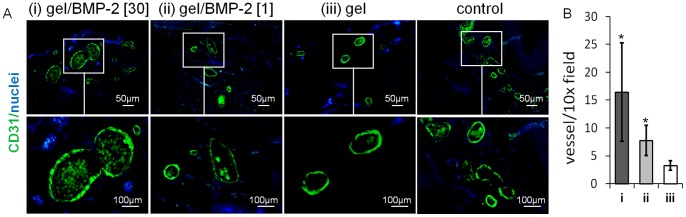
Angiogenesis within the new bone formed. Representative cross sections of cranial bone treated with (i) gel containing 30 µg of BMP-2, (ii) gel containing 1 µg of BMP-2, (iii) gel without BMP-2, or no gel (saline control) at 6 weeks post-injection. Sections were immunostained for CD31 (green). Erythrocytes in vessels are shown by green autofluorescence. Nuclei are in blue. (B) The number of blood vessels per 10x field was quantified. Means ± SD were calculated from n = 3 (3 sections per sample). Comparisons between gel groups and the control group were made by the two tailed Student’s *t*-test for paired samples (*p<0.05).

Blood vessels were visualized by immunofluorescent staining for CD31 (an endothelial-specific marker; Fig. 5A). We observed that the marrow spaces of the new bone formed were rich in functional blood vessels containing erythrocytes (marked by green autofluorescence).The density of the blood vessel were also quantified. As shown in Fig. 5 B, HA hydrogels containing the high dose of BMP-2 had more (16.4±8.8) blood vessels, as compared to the gel with the low dose of BMP-2 (15.8±7.1) and the gel alone (3.3±0.8). Therefore, 30 µg as well as 1 µg of BMP-2 in the hydrogels significantly increased angiogenesis in new bone when compared to hydrogel alone.

## Discussion

Our growing knowledge about stem cells, morphogens, biomaterials, and delivery systems suggests an incredible number of possible combinations for the development of new treatments for non-union fractures, traumatic bone defects, and spinal fusion [20]. In order to evaluate the potential of these new treatments, it has become critical to be able to test them rapidly with standardized preclinical models. This need applies especially to emerging strategies that do not yet have specified applications but that require initial validation.

The development of a standardized model in rats that is reproducible and easy to perform would be an important contribution to tissue engineering and orthopedic research. In the current study, we present a subperiosteal cranial model in rats that was developed on the basis of our previous observations and information in the literature, with the goal of producing a rapid, easy, and reproducible model. We chose the cranial subperiosteal region for this model, since it is easily accessible and because the injection can be visually controlled (Video S1). Because the surgery is based on a single injection and the whole intervention does not exceed 15 min, a single operator without surgical experience can perform the intervention. Moreover, the minimally invasive surgery involved also decreased risk of postoperative infection, experimental failure [27], and morbidity/mortality. Thus, the fact that the surgery is minimally invasive is important to consider in regards to ethical and cost-effectiveness issues, since with currently used animal models of the lowest invasiveness such as the rat calvarial defect model, the morbidity/mortality variable is about 5–10% [28]. In addition, the cranial location offers the advantage to preventing self-injuries or injuries by other rats housed in the same cage [29]. Injection into the periosteal space allows the maintenance of the construct *is situ* (Fig. 1F) and prevents its leakage into the surrounding soft tissues. Moreover, the subperiosteal space is particularly relevant for direct study of the influence of a treatment on mesenchymal stem cells, fibroblasts and other precursor cells, since the periosteum is a source of progenitor cells [21], [22].

Among the currently known regenerative medicine strategies for bone, the use of scaffolds loaded with bone BMP-2 is one of the most promising. Clinically available, BMP-2 is involved in the recruitment and differentiation of bone progenitor cells at the site of bone injury, and it triggers new bone formation [30]. Thus, for the development of our model, we used BMP-2 as an osteoinductive factor. In order to deliver this growth factor, we used the well-established HA hydrogel as an injectable biomaterial standard, because HA scaffolds have been shown to be very efficient as a BMP-2 delivery carriers for BMP-2 in various animal models [12], [14], [31]. For example, we have previously shown that the encapsulation of BMP-2 in HA hydrogel resulted its sustained release *in vitro* for almost a month. This efficient encapsulation was corroborated by potent *in vivo* bone formation at an ectopic site in a rat [12] and calvarial defects in mini-pigs [14]. We decided to use a 6-week time point, because both ectopic and calvarial defect models in the rat are usually stopped within at 4 to 8 weeks [15], [32], [33]. Thus, we believe that a 6-week time period is ideal for evaluating the efficacy of various treatments.

Using this cranial subperiosteal model, we found a significant correlation between the dose of BMP-2 delivered and the amount of new bone formed. This correlation demonstrated the reproducibility of the model and its potential for use in the evaluation of the tretament efficacy. The same type of relationship was observed in a previously published study using a subperiosteal mandibular model in the rat [6]. Using three animals per group, we found low standard deviations for the new bone volume formed in each groups (Fig. 2 B), indicating that the variability of the surgical intervention was low. Furthermore, we found remarkable more and thicker trabeculae with smaller trabecular separation in the bone induced by the gel with high BMP-2 compared to the bone formed by the gel with los BMP-2 and the gel only (Fig. 2C–E). This account for the trabecular thickening and reduction of the trabecular space due to a bone ingrowth.

In addition to the micro-CT evaluation, we used histological staining to further characterize the new bone tissue formed with this model, using various histological techniques. First, we assessed the network of trabecular structure, since it is well accepted that continuity and connectivity of bone trabeculae are important for the transmission of functional forces in human body. For example, in osteoporosis, spontaneous fractures occur when trabeculae in the long bones are resorbed and display disrupted connectivity [34]. As expected, animals treated with the highest dose of BMP-2 displayed new bone tissue with a dense network of trabeculae (Fig. 3). In contrast, the animals treated without BMP-2 or with a low dose of BMP-2 had less extensive trabeculae. As another marker that appears at the stage of bone formation, we detected osteocalcin (OC) within the new bone that was formed. Under all conditions, we were able to detect OC at the sites at which new tissue ingrowth was identified. However, the highest signal was shown in the bone tissues induced by the gels containing 30 µg of BMP-2 (Fig. 4). Furthermore, as another indication of the quality of the new bone formed, we examined the assembly of collagen fibers (Fig. 4). We found collagen fibers of higher organization in the group containing the highest concentration of BMP-2. Finally, we addressed the amount of angiogenesis within the newly formed tissue, since the formation of functional blood vessels plays a pivotal role in skeletal development and bone fracture repair, and inadequate vascularization delays bone graft regeneration [35]. We observed that the marrow spaces of the new bone formed were rich in blood vessels containing erythrocytes (Fig. 5). The higher density of blood vessels was shown in the ectopic bones induced by the high BMP-2 compared to the low BMP-2 and the gel alone. In fact, it was previously reported that BMP-2 does not only stimulates osteogenesis but also enhances angiogenesis [36]. Importantly, in contrast to a previous ectopic bone study in the rat, we did not observe any remaining gels in any of the groups which could be explained by the differences in the local cellular enzymatic activities in the two locations locations used [37].

As a mechanism for bone formation in our model, we propose the following: (1) The progenitor cells from the calvarial bone and from the periosteum migrate toward the BMP-2 gradient in response to its chemotactic activity [38]. The cells adhere to the gel and penetrate its outer surface. (2) They start to differentiate into bone cells and produce organic matrix composed mainly of collagen fibers and non-collagen proteins such as OC. (3) Angiogenesis occurs within the newly formed tissue and contributes to the formation of a bone marrow-like tissue. The vessel network in the new bone is formed from sprouting capillaries existing in the periosteal space, which are subsequently expanded and then mature [39]. (4) The bone-like tissue forms an initial shell of bone that, with time, transforms into a mineralized bone extending toward the center of the scaffold and replacing the gel [40].

The robust bone formation in the animals receiving the high concentration of BMP-2 could be explained by the rapid initial release of some of the BMP-2, while the amount trapped in the scaffold could stimulate later differentiation. In case of the gel with low-dose of BMP-2 it is likely that most of the protein is released at early stage. Similar sequences of bone formation have been observed in an ectopic model after 4 weeks [31] and 8 weeks with the same HA hydrogel in our previous studies [12]. It is also of interest to us to investigate whether and how the excess of bone induced by BMP-2 undergoes remodeling. The excessive bone growth may affect the external contour of the bone, especially in the craniofacial area, and may also affect surrounding structures and organs [14]. Because this model, as an orthotopic model, is suitable for studying osteoclastic resorption in the vicinity of bone, we will complement the dose-response study with an evaluation of the earlier and later time points in a future study.

Like other animal models for bone regeneration, this model has some inherent disadvantages. Rats have quite distinct bone anatomy from that of humans, because they do not have a Harversian system [4]. Therefore, the minimum dose of BMPs necessary to induce consistent bone formation is higher in humans than in rodents [41]. However, the models based on the rat offer the possibility of testing strategies involving non-autologous cells and even xenogenic cells, because athymic rats are available [42]. Another limitation is that this model is not based on a defect, whereas, except for spinal fusion surgery, most clinical applications for bone regeneration tend to be for repair of defects where the availability or supply of bone precursor cells may be less than in just below the periosteum. The defect models have a larger negative impact on the animals’ well-being. In contrast to the defect models in the rat, the model presented here is much faster and easier to perform, and it is certainly more clinically relevant than the ectopic bone model, since the formation of bone occurs in an orthotopic site. In addition, since it is based on a single injection at a specific site, this model is nearly non-invasive and highly reproducible (low standard deviation in the groups) that can lead to a reduction in the number of animals during the course of the study. Consequently, this model is in accord with the principles of the 3 Rs (refinement, reduction, replacement) [43] and, at the same time, allows the possibility of conducting large initial screenings of bone-inducing constructs. Moreover, since HA gels and BMP-2 has been already used in the patients [44], [45], we considered future clinical translation of the construct presented in the study. However, we must be aware that the complexity of human body requires that the final judgment of certain therapy utility and its specific application can be made only after a clinical trial with a long follow-up.

## Materials and Methods

### 1. Protocol

The surgical protocol was approved by the Local Animal Committee of Uppsala University, Sweden (approval no. 222/10). Housing and experiments were performed accordingly to the European Community Council Directive (86/609/EEC). Twelve adult male Sprague-Dawley rats (Taconic M&B, Lille Skensved, Denmark) weighting approximately 250–300 g were housed in a specialized animal facility with an adjusted conditions such as temperature 22–24°C, relative humidity 30–60% and light/dark hours schedule: 12/12. The rats were maintained two per cage and with *ad libitum* access to water and standard rodent diet. Individual body weight was recorded prior to intervention. Animals were acclimated for 10 days prior to surgery. Special attention was given to perform each procedure with the same order and time interval.

### 2. Injectable Construct

Recombinant human BMP-2 (InductOs® Pfizer, former Wyeth Europe) delivered as a lyophilized powder in a formulation buffer containing 2.5% glycine, 0.5% sucrose, 0.01% Polysorbate 80, 5 mM NaCL and 5 mM L-glutamic acid, was reconstituted at concentration of 1.5 mg/mL according to the manufacturer’s instructions by the addition of deionized water and stored at 4°C. The lyophilized form of hydrogel components were synthesised as described elsewhere [12]. Briefly, HA aldehyde (HA-al) and HA hydrazyde (HA-hy) derivatives were dissolved at 16 mg/mL in PBS. HA-hy mixed or not with BMP-2 was loaded into a one 1 mL sterile Luer-lock syringe (Bectron Dickens Medical) and connected by a Luer-lock adapter (Qosina) to another 1 mL syringe loaded with HA-al. The final concentration of BMP-2 was 150 µg or 5 µg per 1 mL of gel. The solutions were mixed 30 times back and forward for 30 s at a room temperature. The gelation time was approximately 1 min.

### 3. *In vivo* Experiment

#### 3.1 Experimental design

Rats were randomly placed in four groups (n = 3) that were treated with 200 µL of the following constructs: (i) HA gel with 30 µg of BMP-2 (ii) HA gel with 1 µg of BMP (iii) gel without BMP-2 and (iv) saline. After 6 weeks, animals were sacrificed by CO_2_ asphyxiation. The area of injection with 1 cm of neighboring bone was collected from the skull. The samples were fixed in 4% paraformaldehyde in PBS at pH 7.4 for at least 48 h at room temperature and stored in 70% ethanol until further analysis.

#### 3.2 Surgical procedure

All surgical procedures were carried out under strictly aseptic and antiseptic conditions. Rats were anesthetized with isoflurane (Forene®, Abbott Scandinavia). After an induction with 5% isoflurane in a small induction chamber, animals were placed in a prone position and anesthesia was maintained with 2% isoflurane in oxygen delivered via a facemask. Once sedated, the rats were shaved and the cranial surface was disinfected with iodine solution and alcohol. The periosteum of frontal bone was exposed by a midline skin incision of approximately 1 cm long, using a surgical blade. A blunt needle (21G) attached to one 1 mL syringe was inserted under the bone periosteum at 45° to the frontal bone surface. The needle was shifted for 15 mm towards the face direction. The periosteum was carefully elevated by the tip in left and right directions. A volume of 200 µL injectable construct or ∼40 µL saline was placed into the subperiosteal space. This amount of saline was used in the previous study where the solution was placed in the periosteal space of the mandibular bone in the rat [6]. In order to avoid the risk of the periosteal contraction we used the same dose of saline. Then, the wound was carefully closed by suturing the skin (4-0 suture, Ethilon™, Ethicon) and there was no need for suturing the periosteum. The intervention was shown in a video file (Video S1). Postoperative pain was managed by the administration of 0.05 mg/kg buprenorphine (Temgesic, Schering-Plough) subcutaneously. Animals presented no sign of pain, distress or infection. Rats were allowed to move freely and were monitored daily.

### 4. Bone Evaluation

#### 4.1 Bone volume calculation

Following sacrifice, the clavariae were resected using an oscillating autopsy saw. Micro-CT analyses were performed using the Skyscan 1072 micro-CT imaging system (Skyscan, Kontich, Belgium) after placing the samples vertically onto the sample holder. Micro-CT images were acquired with a spatial resolution of 18.88 µm (X-ray Source 100 kV/98 kA; Ex posure Time 3.9 sec; Magnification 15X; 1 mm filter applied). Then, using NRecon V1.4 (SkyScan), a cone beam reconstruction was performed on the projected files. Finally, 3D-reconstructions of the samples were obtained (3D-DOCTOR 4.0, Able Software Corp, Lexington, MA). Analysis was performed in the same manner for each rat with a volume of interest corresponding to the injection area. Average bone volume was calculated as bone volume/total tissue volume (BV/TV, mm^3^). In order to determine correlation between BV/TV and the dose of BMP-2, we performed non-parametric Spearman correlation coefficient. Furthermore, the following structural parameters were estimated: trabecular number (TbN,/mm^2^), trabecular thickness (TbTh, mm), trabecular separation (TbS, mm) and porosity (P, %). The parameters TbN, TbTh, TbS and P were tested for normality by the Shapiro-Wilks test. As the data were compatible with a normal distribution, the two-tailed Student’s *t*-test for paired samples was used in the comparison between groups [46]. The standard deviation (SD) was calculated for n = 3. The differences at P<0.05 was considered as statistically significant. The software SAS version 8.2 was used.

#### 4.2 Bone morphology observation

After micro-CT analysis, the cranial bone specimen was completely decalcified using an electrophoresis system (Tissue-Tek Miles scientific, Histolab, Göteborg) with formic acid. Following paraffin processing, 5 µm thick cross-sections were cut with microtome (Thermo Microm HM 355) and stained. For morphological evaluation, the cross sections were stained with Hematoxylin and Eosin (H&E, Histolab, Göteborg). Sections were photographed with a digital camera connected to an optical microscope (Eclipse TE 2000U, Nikon). For collagen observation, the sections were stained with Sirius red (Fluka) [47] and photographed on the polarized light microscope (Leica DM2500 P).

#### 4.3 Expression of angiogenic and osteogenic markers in bone

Immunofluorescence stainings were performed on paraffin sections to visualize osteocalcin (clone FL-100, Santa Cruz Biotechnology) and CD31 (Abcam, ab28364). The sections were deparaffinized, dehydrated in increasing ethanol solutions (ethanol 100-96-80-70-50%, 2 min each), washed in PBS (3×5 min), incubated in a Target Antigen Retrieval Solution pH 9 (Dako) at 95°C for 20 min, and washed with PBS. After blocking in 12% bovine serum albumin (BSA) solution for 1 h at room temperature, the slides were incubated with primary antibody (anti-OC antibody diluted 1∶50, and anti-CD31 antibody diluted 1∶50) overnight at 4°C. Sections were then washed 3 times with PBS and blocked for additional 15 min with 12% BSA in PBS. To visualize the primary antibody, TRITC or FITC-conjugated polyclonal swine or goat F(ab′)_2_ fragments directed to rabbit immunoglobulins (Dako) were added to the sections. Slides were washed 3 times for 5 min in PBS. Sections not treated with the primary antibody were used as a negative control. Finally, the slides were incubated for 5 min in PBS containing 1 µg/mL of Hoechst 33342 (Sigma-Aldrich), washed again with PBS, and mounted with Dako mounting solution (Dako). Pictures of immunofluorescence staining were taken with a DXM1200F digital camera connected to a Nikon Eclipse TE2000-U inverted microscope. The device was equipped with Hoechst 33342, FITC, and TRITC filter sets (Nikon AG; Software: Nikon ACT-1 vers. 2.70). Images were processed with Photoshop 7.0 (Adobe Systems Inc). Instead of qualitative analysis, the density of blood vessels (number of blood vessels per 10x field) was calculated. The standard deviation (SD) was calculated from n = 3 (3 sections per sample). Comparisons between gel groups and the control group were made by the two tailed Student’s *t*-test for paired samples (*p<0.05).

### Conclusions

The evaluation of tissue engineering concepts for bone takes a long time and requires relevant animal models that are suitable for large screening. There is currently no orthotopic model in common use that is cost-effective, easy to perform, reproducible, and employs small animals. As a solution, we presented here a minimally invasive subperiosteal rat model that can be a suitable and relevant tool for the initial screening of regenerative medicine strategies. The model offers a promising approach, even for investigators with little experience in surgery. We have demonstrated that this model provides a stable measuring tool for assessing the biocompatibility and bioactivity of a tissue engineering construct. The outcomes can be rapidly assessed, by correlating quantitative micro-CT data with histological analysis. We believe that this new technique can be used as an alternative to the defect models and can serve as a powerful tool for evaluating regenerative medicine strategies and their mechanisms of action.

## Supporting Information

Video S1 **The video presents a new subperiosteal model in the cranial bone of the rat.** First, the anesthetized animal is placed in a prone position. Further, anesthesia is maintained with 2% isoflurane in oxygen delivered via a facemask. The cranial surface is shaved and disinfected. A surgical blade is used to expose the periosteum of frontal bone by a midline skin incision of approximately 1 cm long. Next, a blunt needle (21G) attached to one 1 mL syringe is inserted under the bone periosteum at 45° to the frontal bone surface. The needle is shifted for 15 mm towards the face direction. The periosteum is carefully elevated by the tip in left and right directions. Then, a volume of 200 µL injectable construct is placed into the subperiosteal space. Finally, the wound is closed by suturing the skin (4-0 suture, Ethilon™, Ethicon).
(ZIP)
